# Evaluation of ES-derived neural progenitors as a potential source for cell replacement therapy in the gut

**DOI:** 10.1186/1471-230X-12-81

**Published:** 2012-06-26

**Authors:** Valentina Sasselli, Maria-Adelaide Micci, Kristen M Kahrig, Pankaj Jay Pasricha

**Affiliations:** 1 Division of Gastroenterology and Hepatology, University of Texas Medical Branch, Galveston, TX, USA; 2 Johns Hopkins Center for Neurogastroenterology, Johns Hopkins University School of Medicine, Baltimore, MD, USA

**Keywords:** Embryonic stem cells, Enteric nervous system, Gastrointestinal motility, Stem cell transplantation

## Abstract

**Background:**

Stem cell-based therapy has recently been explored for the treatment of disorders of the enteric nervous system (ENS). Pluripotent embryonic stem (ES) cells represent an attractive cell source; however, little or no information is currently available on how ES cells will respond to the gut environment. In this study, we investigated the ability of ES cells to respond to environmental cues derived from the ENS and related tissues, both *in vitro* and *in vivo*.

**Methods:**

Neurospheres were generated from mouse ES cells (ES-NS) and co-cultured with organotypic preparations of gut tissue consisting of the longitudinal muscle layers with the adherent myenteric plexus (LM-MP).

**Results:**

LM-MP co-culture led to a significant increase in the expression of pan-neuronal markers (βIII-tubulin, PGP 9.5) as well as more specialized markers (peripherin, nNOS) in ES-NS, both at the transcriptional and protein level. The increased expression was not associated with increased proliferation, thus confirming a true neurogenic effect. LM-MP preparations exerted also a myogenic effect on ES-NS, although to a lesser extent. After transplantation *in vivo* into the mouse pylorus, grafted ES-NS failed to acquire a distinct phenotype al least 1 week following transplantation.

**Conclusions:**

This is the first study reporting that the gut explants can induce neuronal differentiation of ES cells *in vitro* and induce the expression of nNOS, a key molecule in gastrointestinal motility regulation. The inability of ES-NS to adopt a neuronal phenotype after transplantation in the gastrointestinal tract is suggestive of the presence of local inhibitory influences that prevent ES-NS differentiation *in vivo*.

## Background

Several enteric neuronal disorders, such as achalasia, Hirschprung disease, congenital pyloric stenosis and pseudo-obstruction, are characterized by the loss or malfunction of critical neuronal subpopulations of the enteric nervous system (ENS) [1,2]. These conditions result in a severe impairment of digestive functions, for which current surgical and pharmacological therapeutic approaches are not very satisfactory, with the exception of pyloromyotomy for pyloric stenosis [3]. Novel approaches for the treatment of enteric neuropathies are therefore needed and in this respect, stem cell-based replacement of dead or malfunctioning enteric neurons is a promising therapeutic tool [4,5].

Multipotent neural stem cells (NSC) isolated from the brain of embryonic mice can be transplanted in the gut of a transgenic model of gastroparesis and promote significant functional recovery as early as 1 week post-grafting [6-8]. However, the therapeutic use of CNS-derived NSC is limited by the scarce availability of donor tissue and the poor ability of multipotent NSC to generate large number of cells for transplantation *in vitro*. Recent evidence has also shown that enteric neuronal progenitor cells (ENPC) can be derived by autologous biopsy and can be grafted in organotypic gut preparations *in vitro*[9]. Nevertheless the consistency of this approach and the functional recovery after ENPC grafting need further investigation.

Embryonic stem (ES) cells can theoretically overcome many of these limitations and represent an alternative approach to neural precursors. ES cells are pluripotent cells derived from the inner cell mass of pre-implantation blastocysts [10]. ES cells can be propagated almost indefinitely in culture as undifferentiated cells but, under appropriate conditions, are capable of differentiating both *in vitro* and *in vivo* into a variety of cell types including neural cells [11,12]. Over the years, several protocols have been developed to efficiently derive specialized neural cell types such as dopaminergic [13-15], serotonergic [16], GABAergic and glutamatergic [17], telencephalic [18], cerebellar [19], motor [20] neurons from ES cells. Experimental approaches include aggregation into embryoid bodies followed by treatment with retinoic acid (RA), selective propagation of ES aggregates in defined media supplemented with growth factors, culture of ES cells on a monolayer or conditioned media of bone marrow-derived stromal cells (PA6) and genetic lineage selection [21,22]. Furthermore, several studies have also shown ES differentiation into neural crest cells, the developmental progenitors of the ENS, by using combination of defined factors in culture [23-26]. From a therapeutic perspective, therefore, it is of great interest to know whether gut environmental signals can instruct ES cells to differentiate into locally appropriate neuronal types and whether these signals can be further analyzed to improve post-grafting success. Accordingly, the aim of this study was to investigate the effects of putative differentiating cues derived from the ENS and associated gut tissue on ES cells both *in vitro* and *in vivo*.

## Methods

All experimental animal protocols were approved by the Institutional Animal Care and Use Committee at the University of Texas Medical Branch (Galveston, Texas) in accordance with the guidelines provided by the National Institute of Health.

### Mouse ES cells culture

Mouse ES cells from 129/Ola strain (a kind gift of Dr. Jie Du) were maintained for no more than 25 passages in feeder-free conditions on gelatin coated flasks (bovine gelatin from Sigma-Aldrich, flasks from Nunc), in Glasgow’s modified Eagle’s medium (Sigma-Aldrich, St. Louis, MO) supplemented with 2 mM L-glutamine (Invitrogen Corp., Carlsbad, CA), 1 mM sodium pyruvate and 1X solution of non-essential amino acids (both from Sigma-Aldrich, St. Louis, MO), 10% fetal bovine serum (FBS; HyClone), 50 μM 2-mercaptoethanol (Sigma-Aldrich, St. Louis, MO), 100 μg/ml Primocin antibiotic (Amaxa Inc.) and 1000 U/ml LIF (ESGRO, Millipore). ES cells were sub-cultured using 0.05% trypsin/0.02% EDTA (Invitrogen Corp., Carlsbad, CA) in phosphate buffered saline (PBS) and plated at 25,000-30,000 cells cm-2 in freshly gelatinized flasks.

Alkaline phosphatase assay was carried out using the Alkaline Phosphatase Detection Kit (Millipore) according to manufacturer’s instructions.

#### Induction of neurospheres from mouse ES cells

ES cells were trypsinized and plated at a density of 30,000 cells cm-2 onto non-coated flasks in neurobasal medium containing 2 mM L-glutamine, B27 supplement and 100 U/ml penicillin-streptomycin (NB27 medium; all reagents from Invitrogen Corp.), plus 1000 U/ml LIF, 10 ng/ml FGF2 and 50 ng/ml EGF (Promega). Cells were cultured for 7 days as floating multicellular aggregates termed ES-derived neurospheres or ES-NS.

#### Isolation and *in vitro* culture of mouse CNS-derived neural stem cells (CNS-NSC)

Cell culture reagents were obtained from Invitrogen Co. (Carlsbad, California) except where noted. Staged-pregnant female GFP mice (TgN(GFPU)5Nagy, Jackson Laboratory, Bar Harbor, Maine) at embryonic day 15 (E15) were anesthetized with sodium pentobarbital (70 mg/kg, i.p.) and a midline incision was made to expose the embryos. The brains of embryonic mice were removed and the subventricular zone (SVZ) dissected from each brain hemisphere. The tissues were washed in Ca^2+^- and Mg^2+^ -free Hank’s Buffered Salt Solution (HBSS) and digested using a combination of dispase type I (0.1%) and trypsin (0.005%) for 10 minutes at 37°C. After digestion, a cell suspension was obtained by gentle trituration, pelleted and resuspended in Neurobasal medium containing B27, 2 mM glutamine and penicillin-streptomycin (NB27), plus 20 ng/ml fibroblast growth factor (bFGF) and 20 ng/ml epidermal growth factor (EGF) (Promega, Madison, Winsconsin).

#### Differentiation of mouse ES-NS and CNS-NSC

To assess differentiation, multicellular aggregates were dissociated by incubation with Accutase (Innovative Cell Technologies, Inc., San Diego, CA) at 37°C for 10 minutes. Single cell suspensions were plated onto poly-ornithine (Sigma-Aldrich, St. Louis, MO)/mouse laminin (Invitrogen Corp, Carlsbad, CA) coated plates (BD FalconTM, BD Biosciences, San Jose, CA) in NB27 medium without growth factors and cultured for 7 days.

#### Co-culture with muscularis externa-myenteric plexus

Adult C57BL/6 J and TgN(GFPU)5Nagy mice (3-5 week old males, Jackson laboratories) were used. The TgN(GFPU)5Nagy mice have the Enhanced Green Fluorescent Protein (EGFP) driven by chicken beta-actin promoter and CMV intermediate early enhancer which drives GFP expression ubiquitously in all tissues. Mice were euthanized by cervical dislocation. The small intestine was dissected in pieces of 1 cm length and placed over a glass rod. A small incision was made and muscle layers with the adherent myenteric plexus were peeled off using cotton swab. The majority of preparations contained only longitudinal muscle and myenteric plexus and therefore we referred to them as LM-MP. LM-MPs were cultured at 37°C in 5% CO2 for 24 hrs in DMEM containing 10% FBS and 100U/ml penicillin-streptomycin (all reagents from Invitrogen Corp.). ES-NS were dissociated and plated onto poly-ornithine/laminin coated plates at a density of 2000 cells cm-2 in NB27 medium supplemented with 1000 U/ml LIF, 10 ng/ml FGF2 and 50 ng/ml EGF. Cells were cultured for 18 hrs before placing the LM-MPs into a 0.4 μm microporous insert (Corning Inc., Corning, NY, USA) inside each well (5 pieces per 24 mm-diameter insert). Co-cultured ES-NS were maintained in NB27 medium without growth factors and cultured for 10 days. Freshly prepared LM-MPs were placed in the inserts after 5 days. Control ES-NS were cultured in the same conditions without LM-MPs.

### Flow cytometry analysis

Cells were dissociated using Accutase digestion according to manufacturer’s instructions (Innovative Cell Technologies, Inc., San Diego, CA) and collected by centrifugation. 5 x 10^5^ – 1 x 10^6^ cells were fixed in ice-cold 4% paraformaldehyde for 15 minutes at room temperature (RT). Cells were then blocked and permeabilized for 5 minutes in PBS containing 0.1% Triton X-100 and 2% normal goat serum (NGS). Incubation with Ki67 antibody diluted in PBS containing 2% NGS was carried out for 30 minutes at RT, followed by incubation with secondary antibody for another 30 minutes (see Table 1 for a list of antibodies used). Cells were resuspended in 500 μl of PBS containing 2% NGS and processed with the analytical Flow Cytometer FACSCanto (Becton-Dickinson, Franklin Lakes, NJ USA).

**Table 1:** List of primary (A) and secondary (B) antibodies used for Immunofluorescence (IF), Flow Cytometry (FC) and Western blot (WB) analysis

**A.**				
***Primary Ab***	***Host***	***Dilution***	***Specificity***	**Source**
Ki67	Rabbit	1:100 FC	Proliferating cells	Abcam
Nestin	Mouse	1:2000 WB, IF	Neural progenitor cells	BD
βIII-Tubulin	Mouse	1:2000 IF; 1:5000 WB	Neurons	Promega
	Chicken	1:2000 IF	Neurons	Aves Laboratories
PGP 9.5	Rabbit	1:2000 WB, IF	Neurons	Chemicon
Peripherin	Rabbit	1:2000 WB, IF	Neurons	Chemicon
MAP-2	Chicken	1:2000 WB, IF	Neurons	Aves Laboratories
nNOS	Mouse	1:2500 WB	Neurons	BD
	Rabbit	1:200 IF	Neurons	Zymed
GFAP	Rabbit	1:2000 IF 1:5000 WB	Glial cells	DAKO
α-SMA	Rabbit	1:1000 IF 1:5000 WB	Smooth muscle cells	Abcam
GAPDH	Mouse	1:10000 WB	Loading control	Abcam
**B.**				
***Secondary Ab***	***Host***	***Dilution***	***Source***	
Alexa 633 anti rabbit	Goat	1:200 FC	Molecular Probes	
Alexa 594 anti mouse or rabbit	Goat	1:200 IF	Molecular Probes	
Alexa 488 anti mouse or rabbit	Goat	1:200 IF	Molecular Probes	
HRP conjugated anti mouse or Rabbit	Goat	1:20000 WB	Zymed	

### Real time PCR analysis

Total RNA was extracted from cells using the TRIzol kit (Invitrogen Corp.) according to manufacturer’s instructions. First-strand cDNA was then generated using TaqMan Reverse Transcription Regents (Applied Biosystems, Foster City, CA, USA). Quantitative real-time polymerase chain reaction (PCR) was carried out in multiplex using the PTC 200 Peltier Thermal Cycler equipped with Chromo 4 Continuous Fluorescence Detector (MJ Research Waltham, MA, USA). PCR was performed using GeneAmp PCR kit and AmpliTaq Gold polymerase (Applied Biosystems) with the following cycling conditions: 2 minutes at 50°C, 10 minutes at 95°C, followed by 40 cycles of 15 seconds at 95°C, 1 minute at 60°C and fluorescence plate reading. The following sequence-specific primers and probes were designed using Primer Express software 2.0 (Applied Biosystems): β-III tubulin: Fw GGTCTGGCGCCTTTGGA, Rv AGTTGTTGCCAGCACCACTCT, probe Cy5-CCTATTCAGGCCCGACAACTTTATCTTTGGT-IBRQ; PGP9.5: Fw GCTTCGCCGACGTGCTA, Rv TTGCTTTTTCCTGAAGTTTTCATG, probe Cy5-CTGCTGCTCCTGTTTCCCCTCACG-IBRQ; peripherin: Fw GCATCTCAGTGCCGGTTCAT, Rv TGGGACTCTGTCACCACCTTCT, probe TexRed-TTGCCTCTCTAAGTTTAAAGACGACTG-IBRQ; nNOS: Fw GGGAAACTCTCGGAGGAGGA, Rv TGAGGGTGACCCCAAAGATG, probe 6FAM-CGTGGTACCGGTTGTCATCCCCTCAG-TAMRA; GFAP: Fw GAGGAGGAGATCCAGTTCTTAAGGA, Rv GCCTCGTATTGAGTGCGAATC, probe 6FAM-CCAGACCTCACAGCGGCCCTGA-TAMRA; α smooth muscle actin: Fw GAGACTCTCTTCCAGCCATCTTTC, Rv TGATGCTGTTATAGGTGGTTTCGT, probe 6FAM-ATGCCCGCTGACTCCATCCCAA-TAMRA. Ribosomial RNA 18 s (primers and VIC-conjugated probe from Applied Biosystems) was measured as a reference gene. Cycle Threshold (CT) values were quantified using Opticon Monitor 2 software (MJ research) and normalized calculating the mRNA level of each probe (x) relative to the amount of 18 s mRNA (reference gene) as follow: mRNA(x) = 2 CT (18 s) – CT (x) x 100. Mouse brain cDNA was used as positive control for all primers and probes and NO-RT reactions were used as negative control to check for genomic DNA contaminations.

### Western blot analysis

Cells were dissociated and collected in pellets, which, after incubation overnight at -80°C, were lysed in ice-cold buffer containing 2% sodium dodecyl sulphate (SDS), protease cocktail inhibitor (Sigma-Aldrich), 1 mM phenylmethylsulphonylfluoride (PMSF), 1 mM dithiothreitol (DTT), 5 mM ethylenediaminetetraacetic acid (EDTA) in 50 mM Tris-HCl, pH 7.4. After centrifugation, the supernatants were collected and protein content was determined (BSA Protein Assay Kit; Pierce). Protein samples were processed for SDS-polyacrylamide gel electrophoresis (PAGE) with Mini-Protean Gel-3 system (Bio-Rad laboratories, Hercules, CA, USA). After electrophoresis, proteins were transferred to Polyvinylidene Difluoride (PVDF) membranes (Bio-Rad). Blots were incubated in blocking buffer (5% non-fat dry milk in TBS containing 0.1% Tween 20; TBST) for 1 hr at RT and probed with primary antibodies (Table 1A) overnight at 4°C and horseradish peroxidase (HRP)-conjugated secondary antibodies (Table 1B) for 1 h at RT. Detection was performed using ECL plus kit (Amersham) and exposure of X-ray films (Bioexpress) then developed with M35A-X-OMAT Processor (Kodak, Rochester, NY, USA). Band intensity was quantified using Optiquant software (Packard, Meriden, CT, USA) and data were normalized to the expression of the housekeeping protein GAPDH.

### Immunofluorescence analysis

Cells were fixed in ice-cold 4% paraformaldehyde for 15 minutes at RT, blocked and permeabilized for 1 hr with PBS containing 0.3% Triton X-100 and 5% NGS. Samples were incubated with primary antibodies (Table 1A) diluted in PBS containing 5% NGS overnight at 4°C and with secondary antibodies (Table 1B) for 1 hour at RT. Sections and slides were cover-slipped with mounting media containing the nuclear stain DAPI (Vector Laboratories, Burlingame, CA). LM-MP preparations were processed similarly with the following modifications: incubation in Pronase (Biomedia) for 5 minutes at RT after fixation, primary and secondary antibody incubation for 72 hrs and 1.5 hrs respectively at RT in PBS containing 1.5% NGS, 0.3% Triton-X100 and 0.01% Na Azide. Samples were examined with an Olympus BX60 microscope (Olympus, Melville, NY, USA) equipped with fluorescence and digital imaging with a cooled CCD camera (Photometrics CoolSNAP, Roper Scientific, Duluth, GA, USA) and Metaview software (Universal Imaging Corp., West Chester, PA, USA).

Cell counts were performed by counting the number of immunopositive cells for a given antibody and expressing the results as number of positive cells versus total cells number (determined using the nuclear counterstain DAPI that forms fluorescent complexes with natural double-stranded DNA and it is used to label the nuclei in all the cells). An independent investigator who was blinded to the treatment counted three regions per slides. A total of 3 experiments were counted.

### Intra-pyloric transplantation

To enable the detection of the cells *in vivo*, dissociated ES-NS cells were labeled with CM-DiI (Molecular Probes, Eugene, OR) according to manufacturer’s instructions. After washing in PBS, the cells were resuspended at a concentration of 50,000 cells/μl in PBS containing 500 μM caspase-1 inhibitor (Ac-YVAD-CMK, Calbiochem, La Jolla, CA, USA) and kept on ice. Adult male C57BL/6 J mice (Jackson laboratories) were anesthetized with 0.4 ml of 6% ketamine/4% xylazine intraperitoneally. A midabdominal incision was made and the pylorus identified. Two microliters of ES-NS suspension were injected bilaterally into the mid-pylorus immediately subserosal using a 22-gauge needle attached to a 10 μl Hamilton syringe.

One week following ES-NS transplantation, mice were deeply anesthetized with sodium pentobarbital (70 mg/kg intraperitoneally), transcardially perfused, and fixed with freshly prepared ice-cold 4% paraformaldehyde in 0.1 M PBS (pH 7.4). The pylorus was removed, post-fixed in 4% paraformaldehyde, and cryoprotected by infiltration in 20% sucrose solution in PBS overnight at 4°C. Tissues were rapidly frozen in O.C.T. embedding medium (Tissue Tek; Sakura, Tokyo, Japan) over dry ice–chilled isopentane (Sigma Chemical Co, St Louis, MO). Frozen serial sections (16 μm thick) were cut on a cryostat (TBS, Durham, NC), placed on Superfrost Plus slides (VWR, West Chester, PA), and stored at -20°C until needed.

### Statistical analysis

RT-PCR and WB data were expressed as mean ± SEM. For comparisons between the groups One Sample *T* Test was used. Statistical significance was assumed if P < 0.05.

## Results

### Characterization of murine ES cells

**Figure 1:**
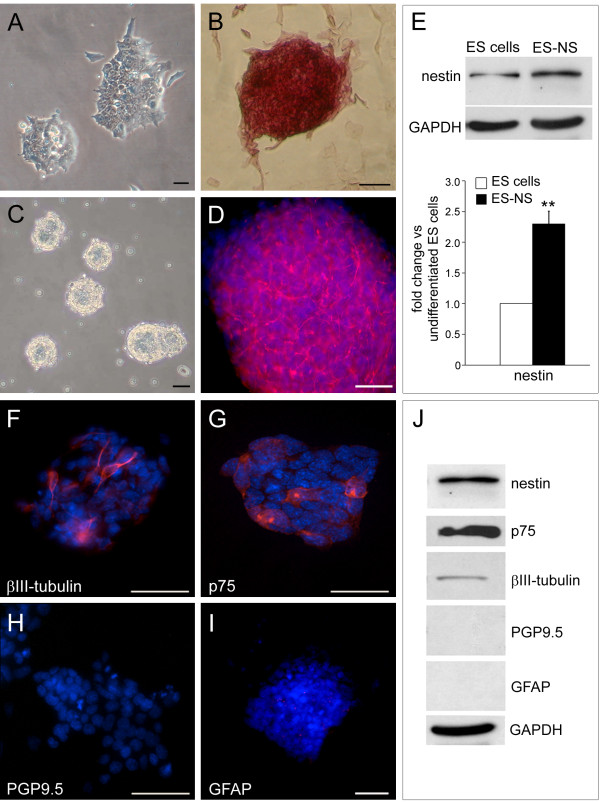
**Characterization of 129/Ola murine ES cells and of ES-derived neurospheres.** (**A**) Phase contrast image of ES cell colonies with the characteristic compact morphology. (**B**) ES cells colonies show a high expression of the stem cell marker alkaline phosphatase. Calibration bars = 25 μm. (**C**) Phase contrast micrograph of ES-derived neurospheres (ES-NS) after 7 days in culture in serum-free medium supplemented with growth factors (NB27 + GF) and (**D**) immunofluorescence analysis showing the expression of the neural stem cell markers nestin (red). Nuclei are counterstained with DAPI (blue). Calibration bars = 25 μm. (**E**) Western blot analysis of nestin expression in ES-NS compared to ES cells grown under regular conditions. n = 3 ** P < 0.01 One Sample *T* test. (**F**-**I**) Immunofluorescence analysis of ES-NS showing the expression of the neural crest stem cell marker p75 (red); of the neuronal progenitor marker βIII-tubulin (red); and the lack of expression of the neuronal marker PGP9.5 (green) and of the glial marker GFAP (red). Nuclei are stained with DAPI and are shown in blue. Calibration bars = 25 μm. (**J**) Western blot analysis of ES-NS.

Characterization of the 129/Ola ES cell line was carried out by investigating common attributes of embryonic stem cells: colony morphology and alkaline phosphatase expression. Murine ES cells grown in feeder-free conditions formed colonies with the typical round and compacted morphology (Figure 1A) and with a high expression of alkaline phosphatase, a marker expressed by embryonic stem cells but not by their differentiated derivatives (Figure 1B) [27].

### Induction of neural progenitors from ES cells

In order to start with an enriched population of neural precursors we have modified existing protocols for generating ES-derived neurospheres [14,15]. ES cells were grown for 7 days into non-coated tissue culture flasks in Neurobasal medium supplemented with the neurotrophic serum-free supplement B27 and the growth factors FGF-2, EGF and LIF. Under these conditions ES cells grow as floating multi-cellular aggregates, which morphologically resemble the neurospheres that are formed by neural stem cells (NSC) isolated from fetal and adult brain (Figure 1C). ES cells show expression of the neural stem cell marker nestin suggesting spontaneous neural differentiation under the culture conditions used (data not shown). This is not unusual and has been previously reported by other investigators [28]. However, the expression of nestin was limited to few ES cells (data not shown) and was significantly increased after neural induction in ES-derived neurospheres (ES-NS) (Figure 1D), with a 2.3 fold increase in protein expression compared to undifferentiated ES cells cultures (P < 0.01, n = 3; Figure 1E).

Some cells within the neurospheres also express the neural crest stem cell marker p75 and the neural progenitor marker βIII-tubulin. On the other hand, the mature neuronal marker PGP9.5 and the glial marker GFAP were not detectable in ES-NS (Figure 1F-I and Figure 1J).

**Figure 2:**
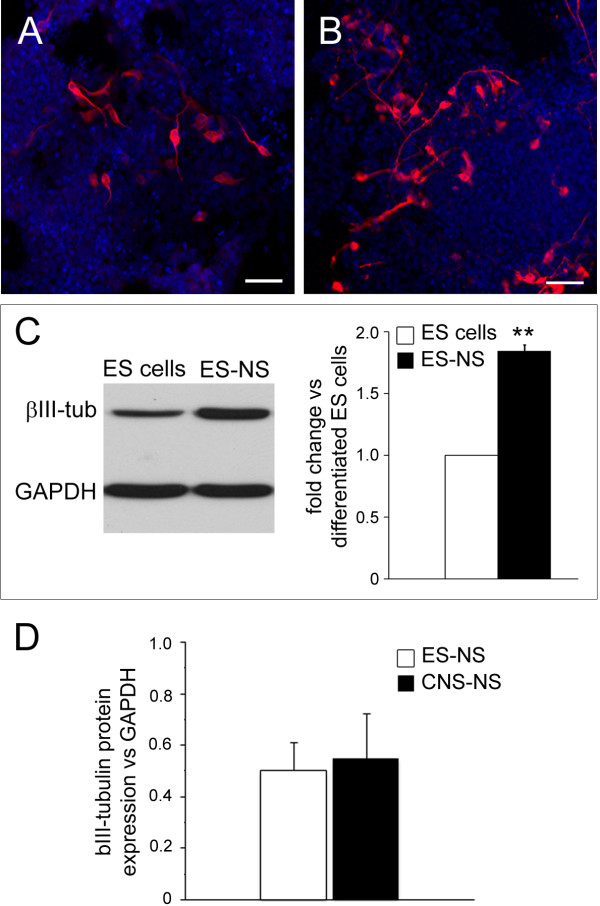
**Neurogenic potential of ES-derived neurospheres.** ES cells (**A**) and ES-NS (**B**) were plated out onto poly-ornithine/laminin-coated plates and allowed to differentiate by culturing them in NB27 medium without growth factors for 7 days. More βIII-tubulin-positive cells (in red) are visible in ES-NS cultures as compared to ES cells differentiated under the same conditions. Nuclei are counterstained with DAPI (blue). Calibration bars = 25 μm. (**C**) Western blot analysis for βIII-tubulin expression in differentiated ES-NS compared to differentiated ES cells. n = 3, **P < 0.01 One Sample *T* test. (**D**) Western blot analysis for βIII-tubulin expression in differentiated ES-NS and CNS-NS. n = 3 for ES-NS and n = 4 for CNS-NSC, P = 0.83, Student’s *T*-test.

To further explore the neurogenic potential of ES-NS, neuronal differentiation of ES-NS was assayed after 1 week of culture in the absence of any growth factor and compared to that of uncommitted ES cells. Under these conditions ES-NS were able to generate neurons identified by a clear uni/bi-polar neuronal morphology (Figure 2A-B) and by the expression of the neuronal marker βIII-tubulin (1.8 fold increase as compared to ES cells; P < 0.01, n = 3. Figure 2C). Moreover, when we compared differentiated ES-NS to cells differentiated from CNS-derived neurospheres (CNS-NS), we found that the expression level of βIII-tubulin measured by Western blot analysis was similar between the two groups (Figure 2D).

Interestingly, ES-NS did not generate astrocyte-like cells as indicated by the lack of expression of glial fibrillary acidic protein (GFAP), a marker of the glial lineage, as assessed by both Western blotting and RT-PCR (data not shown).

### Small intestinal longitudinal muscle-myenteric plexus preparations

**Figure 3:**
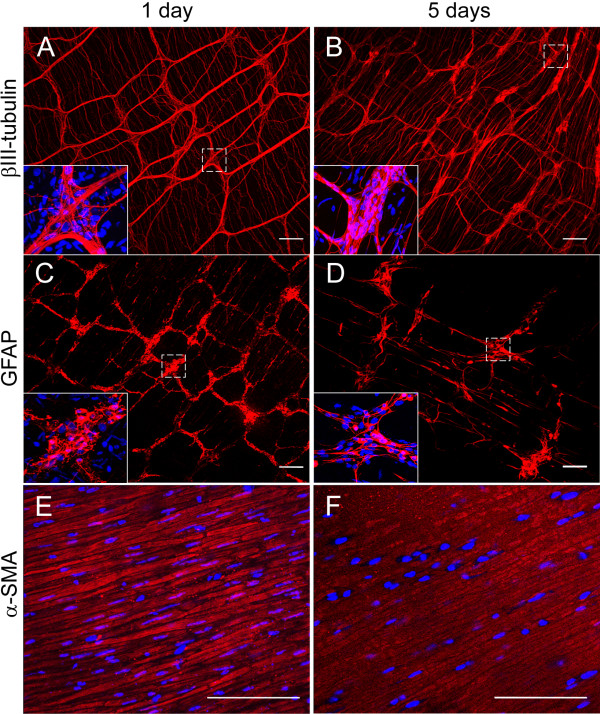
**Time course analysis of small intestine longitudinal muscle – myenteric plexus (LM-MP) preparations.** Immunoreactivity for the neuronal marker βIII-tubulin (red, A-B) and for the glial marker GFAP (red, C-D) reveal a preserved anatomy of the myenteric plexus and its ganglia (insets) in mouse small intestine LM-MP strips maintained in culture in NB27 medium for 5 days (**B**, **D**) compared to fresh preparations (**A**, **C**). Similarly, the persisting reactivity for α-smooth muscle actin, α-SMA (red, E-F) shows the longitudinal muscle still intact after 5 days in culture (**F**, fresh preparation control in **E**). Nuclei are counterstained with DAPI (blue). Calibration bars = 50 μm.

To investigate the effects of putative gut factors on the differentiation of ES-NS we established an *in vitro* co-culture protocol. The procedure involves the organotypic culture of strips of longitudinal muscle and the adherent myenteric plexus (LM-MP) derived from the small intestine of adult mice. LM-MP tissues were placed into the inside compartment of transwells separated from the ES-NS by a microporous membrane (0.45 μm pore size) that allows the exchange of soluble factors but not of cells. The maximum length of time for co-culture was defined as the approximate time needed for cells to reach confluency (10 days). To prove the effectiveness of this system we performed preliminary experiments in which LM-MP preparations were obtained from TgN(GFPU)5Nagy mice. No GFP positive cells were found in the compartment containing the ES-NS cells after 10 days of co-culture (data not shown), proving that only soluble molecules are exchanged between the two sides of the membrane. LM-MP preparations were also tested by immunoreactivity for βIII-tubulin, GFAP and α-smooth muscle actin (α-SMA) during the co-culturing period, which showed integrity of both the neural plexus and smooth muscle for at least 5 days (Figure 3) and attested to their viability. Therefore, in the co-culture experiments, LM-MP strips were changed with fresh preparations at day 5 of the culturing period, in order to avoid tissue degeneration.

### Neurogenic potentials of ES-NS in co-culture with LM-MP

**Figure 4:**
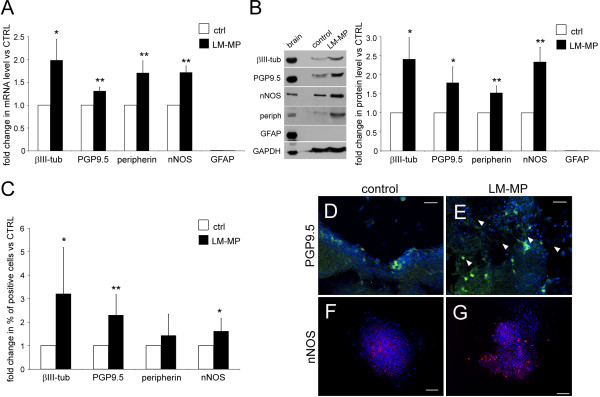
**Co-culture of ES-NS with LM-MP preparations for 10 days leads to a significant increase in neuronal differentiation.** Expression of neuronal and glial markers was assessed by RT-PCR (**A**), Western Blot (**B**) and immunohistochemistry (**C**). The pan neuronal markers βIII-tubulin and PGP9.5 and peripherin, as well as the neuronal subtype marker nNOS were increased both at the mRNA and protein levels in the LM-MP co-cultured group as compared to control (fold change ranging from 1.3 to 3.2). The glial marker GFAP was not detected at this stage of differentiation by any of the analysis performed. (**A**) Changes of mRNA level in ES-NS co-cultured with LM-MP as compared to control cultures (ES-NS without LM-MP). n = 7. * P < 0.05, ** P < 0.01. (**B**) Changes in protein level in ES-NS co-cultured with LM-MP as compared to control cultures. n = 6. * P < 0.05, ** P < 0.01. (**C**) Changes in percentage of positive cells in ES-NS co-cultured with LM-MP as compared to control cultures. n = 3. * P < 0.05, ** P < 0.01. (**D**-**E**) Representative images of immunoreactivity for PGP9.5 (green), a marker for mature neurons, in control cultures and ES-NS co-cultured with LM-MP. PGP9.5 immunoreactivity extends to the neurites (arrows, E) although it is less intense partly due to the difference in focal planes. Scale bar = 25 μm. (**F**-**G**) Representative images of immunoreactivity for nNOS (red) in control cultures and ES-NS co-cultured with LM-MP showing increased proportion of nNOS^+^ cells after co-culture with LM-MP compared to controls. Scale bar = 50 μm. Nuclei are counterstained with DAPI (blue).

When co-cultured with mouse LM-MP, ES-NS showed a significant increase in the mRNA expression of the neuronal markers βIII-tubulin, PGP 9.5 and peripherin as well as of neuronal nitric oxide synthase, nNOS, as compared to control ES-NS cultured without LM-MP (2.0, 1.3, 1.7 and 1.7 fold increases respectively, P < 0.05, n = 7; Figure 4A). This was accompanied by a corresponding increase in protein expression as shown by Western blot analysis (2.4 fold for βIII-tubulin, 1.8 for PGP 9.5, 1.5 for peripherin and 2.3 for nNOS, P < 0.05, n = 6. Figure 4B). The expression of ChAT was not detected in differentiated ES-NS (data not shown). Interestingly, ES-NS did not generate astrocyte-like cells as indicated by the lack of GFAP expression, both at the mRNA and protein level.

**Figure 5:**
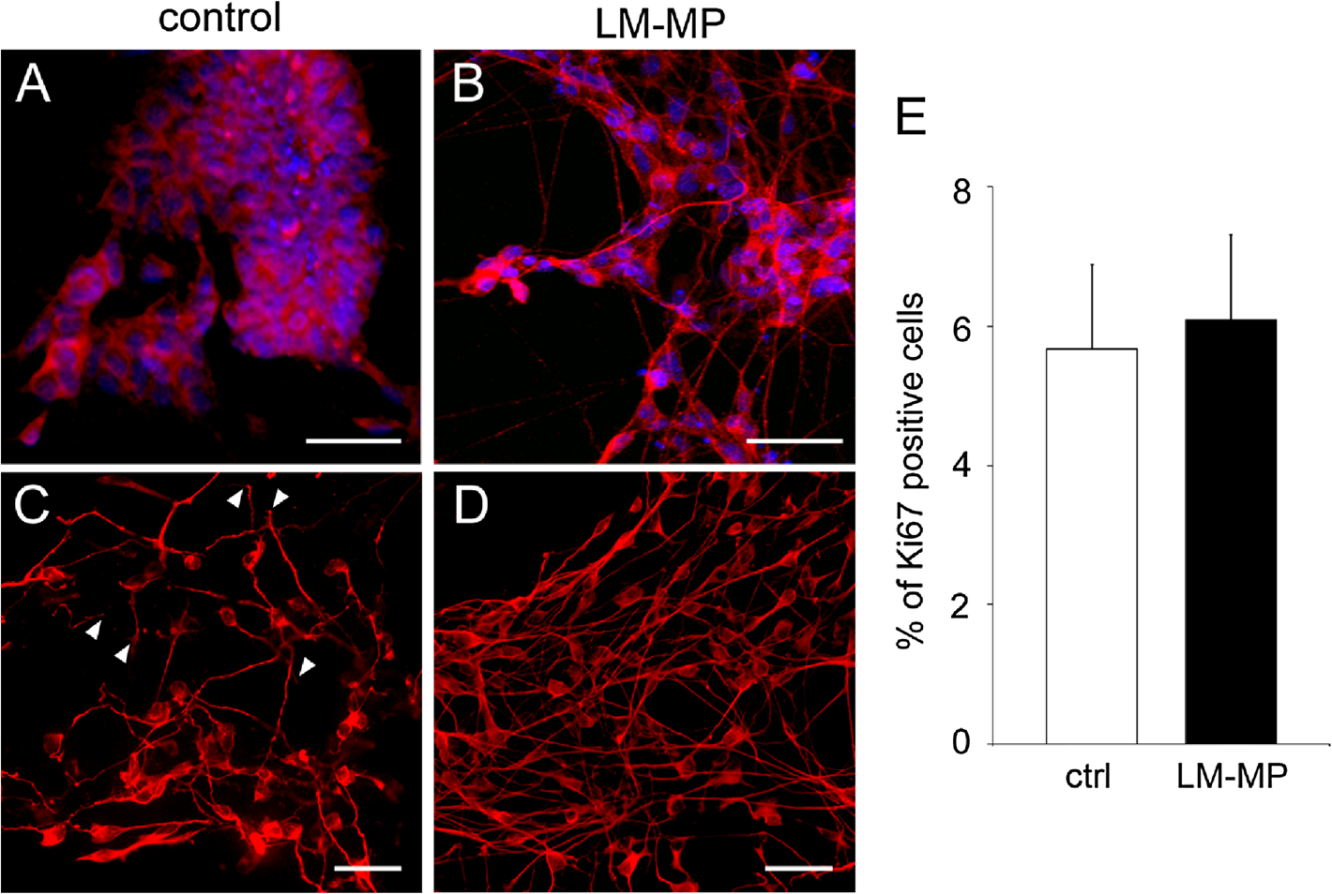
**Differentiated neurons in LM-MP treated ES-NS exhibit a more complex and mature morphology than control neurons.** Morphology of neurons is shown by immunoreactivity for the neuronal markers peripherin (red, A-B) and βIII-tubulin (red, C-D) in ES-NS co-cultured with LM-MP (**B**, **D**) or control (**A**, **C**). Neurons arising from ES-NS exposed to LM-MP show pronounced processes and form a network-like structure, whereas control neurons show either absence of neuritogenesis (**A**) or shorter blunt processes (**C**) white arrowheads. Nuclei are counterstained with DAPI (blue). Calibration bars = 25 μm. (**E**) Analysis of the expression of the cell cycle marker Ki67 by flow cytometry does not reveal any significant change between the two groups. n = 3, P = 0.81, Student’s *T*-test.

Immunofluorescence analysis corroborated these findings, revealing a significant increase of percentages of cells positive for βIII-tubulin, PGP9.5 and nNOS after exposure to LM-MP compared to control cultures (βIII-tubulin 42.3 ± 7.0% vs 23.6 ± 6.3%, PGP9.5 39.9 ± 6.6% vs 21.6 ± 4.7%, nNOS 15.8 ± 3.5% vs 11.8 ± 4.6%, with 3.2, 2.3 and 1.6 fold increases respectively, P < 0.05, n = 3. Figure 4C-D). Moreover, neurons showed a more developed morphology after co-culture with LM-MP than in control cultures as highlighted by immunoreactivity for cytoskeleton proteins such as peripherin (Figure 5A-B) and βIII-tubulin (Figure 5C-D). It has to be noted that in the control group, the number of cells expressing peripherin in relation to the total number of cells in the culture was relatively low (as confirmed by the low signal in the WB analysis shown in Figure 4B). However, the cells that did express peripherin were highly positive by immunofluorescence analysis thus explaining the images that we are showing in Figure 5.

We also demonstrated that increased cell proliferation is unlikely to account for elevated levels of neuronal markers since the percentage of Ki67 expressing cells is similar in the co-culture and control groups (6.1 ± 1.2% and 5.6 ± 1.2% respectively, P = 0.81, n = 3; Figure 5E).

**Figure 6:**
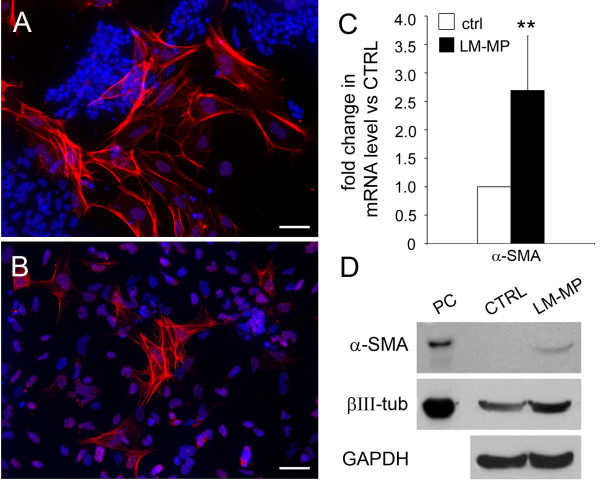
**Smooth-muscle actin expression in ES-NS.** α- SMA immunoreactivity (red) in ES-NS cultured in the presence (**A**) or absence (**B**) of LM-MP. A small percentage of α-SMA positive cells were generated from LM-MP treated ES-NS as compared to the control group where they were rare. Nuclei are counterstained with DAPI (blue). Calibration bar = 25 μm. (**C**) RT-PCR analysis confirmed the increase of α-SMA transcript in LM-MP treated ES-NS as compared to control (n = 6, ** P < 0.01). This result was corroborated by Western Blot analysis shown in panel **D** (40 μg of total protein extract per lane. Loading control GAPDH, Glyceraldehyde 3-phosphate dehydrogenase), indicating increased expression of α-SMA in the LM-MP co-culture group as compared to control where the expression of α-SMA was negligible.

Immunofluorescence analysis also revealed the appearance of a small percentage of cells (less than 1% of the total) positive for smooth muscle marker α-SMA in the co-cultured ES-NS (Fig 6A-B). By Western blot analysis we could detect α-SMA only after co-culture with LM-MP (Fig 6D). This is most likely due to the signal being below the detection limit due to the low number of cells positive for this marker in the control culture. However, RT-PCR confirmed a 2.7 fold increase of SMA expression in the co-cultured group as compared with control (P < 0.01, n = 6; Figure 6C).

### Intrapyloric transplantation of ES-derived neurospheres

**Figure 7:**
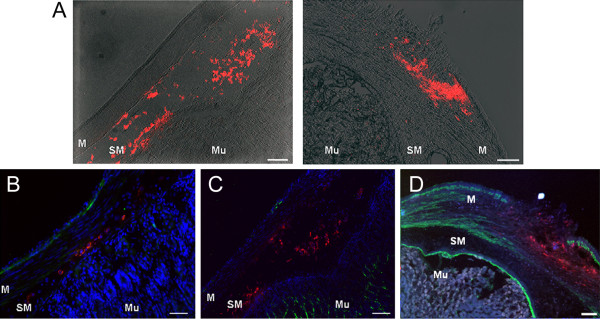
**Intrapyloric transplantation of ES-NS.** (**A**) Grafted DiI-labeled ES-NS (red) after 7 days from transplantation were found mainly in the muscle and submucosal layers. (**B**-**D**) Immunofluorescence staining revealed a failure by the grafted ES-NS to express mature phenotype markers such as peripherin (green, B), GFAP (green, C) and α-smooth muscle actin (green, D). Nuclear staining with DAPI (blue). M, muscle; SM, submucosa; Mu, mucosa. Calibration bars = 25 μm.

To assess preliminary feasibility of ES-derived neurospheres transplantation, we used the experimental protocol of intrapyloric transplantation previously described for CNS-derived neural stem cells (CNS-NS) [6-9]. ES-NS were grafted in the pyloric muscle layer of adult male mice; survival and differentiation were assessed 1 week after transplantation. At the time point chosen grafted cells were found mainly in the muscle and the submucosal layers showing good survival (Figure 7A). On the other hand, immunohistochemistry failed to reveal expression of the neuronal markers βIII-tubulin, peripherin, MAP-2 and nNOS as well as the non-neuronal markers GFAP and α-smooth muscle actin by the engrafted ES-NS (Figure 7 B-D).

## Discussion

Although ES cells can differentiate into neural precursors in the absence of any external influence, only a small percentage of neural cells survive and differentiate under these conditions [29,30]. In this report we have therefore developed a modified protocol to enrich our culture in ES-derived neural progenitors. Using this approach we obtained multi-cellular aggregates that share many properties with neurospheres generated from CNS-derived neural stem cells, namely, sphere morphology, nestin expression and the ability to produce neurons. Moreover, ES-NS express p75, a marker found in neural crest-derived stem cells [31]. Having established a neuronally biased ES population, we next examined the effects of an enteric neuromuscular environment on its further differentiation. It has been shown that co-culture of progenitor cells with mature cells or tissues can drive their differentiation toward desired phenotypes [32,33]. We therefore established a controlled *in vitro* system where ES-NS are co-cultured with strips of small intestine longitudinal muscle and the adherent myenteric plexus (LM-MP). Although the presence of the myenteric plexus in the LM-MP preparation might suppress ES-NS differentiation, our results show that intestinal LM-MP exert a considerable neurogenic effect on ES-NS, as evidenced by the enhanced expression of several neuronal markers, including peripherin, the intermediate filament which is a well established marker of peripheral neurons [34] and the enzyme neuronal nitric oxide synthase (nNOS), responsible for the production of the critical enteric neurotransmitter, nitric oxide [35]. On the other hand, ChAT was not expressed in ES-NS under either co-culture or control conditions. Although our data does not provide an explanation for the lack of ChAT expression, it is interesting to note that nNOS-expressing neurons appear early during ENS development while ChAT-expressing neurons are only detected at later embryonic stages and post-natally [36-38].

The neurogenic effect observed after co-culture of ES-NS with LM-MP could be attributed to either increased proliferation and/or improved differentiation of neural progenitors within ES-NS. Our data show that the number of neurons (PGP9.5 and nNOS positive) is increased after co-culture with LM-MP, but not the number of proliferating cells (Ki67 positive). Moreover, co-culture with LM-MP clearly affects the morphology of neurons produced, which possess longer axons and form a more extensive and organized network as compared to controls. This suggests that soluble factors released from the LM-MP promote survival and differentiation of neural progenitors but not their proliferation. Therefore the main effect in our ES-NS co-culturing protocol is to favor differentiation of neurons.

In addition to inducing neuronal differentiation, we also observed a minor myogenic effect of LM-MP on ES-NS. Previous reports have shown that CNS-derived NS can be induced to express smooth muscle markers *in vitro*[39], on the other hand, neural crest cells (NCC), which give rise to enteric neurons and glia during development, are also able to generate smooth muscle cells in different embryonic compartments [40]. Therefore our findings could be due to an *in vitro* artifact or to a NCC-like potential of ES-NS.

ES-NS failed to express the glial marker GFAP under any of our culture conditions, unlike other studies using ES-derived neural precursors, which have reported the capability of these progenitors to differentiate into both the neuronal and glial lineage *in vitro*[11,14]. The discrepancy between these reports and our results may be due to differences in culture conditions used to produce ES-NS. Alternatively, the fact that neural progenitors have a stage-dependent potential giving rise first to neurons and then to glia during neural commitment [41], may suggest that prolonged culture periods are necessary to generate glial cells. Therefore differentiation of ES-NS for longer periods of time (more than 10 days) and analysis of a more widespread panel of glial markers will need to be performed.

It is well known that the environment plays a key role on the differentiation of ES cells into defined phenotypes. We have developed a robust and informative *in vitro* system where soluble factors released by gut tissues are able to drive neuronal differentiation of ES-derivatives appropriate for the ENS lineage. The nature of these signals has yet to be determined, but may include well-known soluble factors of the gut such as GDNF, NO, TGFβ (respectively produced by glial, neuronal and muscle cells) or other molecules. Further analysis of the LM-MP conditioned medium will help to identify these factors.

Although transplantation of ES-NS in the pyloric muscle layer of wild type mice showed adequate survival of grafted cells at 1 week, no markers of terminal differentiation were noted. This contrasts with many reports that showed successful transplantation of ES-derived neural precursors in the CNS [13-15]. This may be due to the presence of inhibitory factors, either secreted or contact-based that were not captured *in vitro* using isolated LM-MP co-cultures. This difference also probably reflects the vastly more complex environment of the gut wall, consisting of a mixture of epithelium, neurons, glia, interstitial cells of Cajal and smooth muscle, along with resident and circulation-derived immunocytes. These cellular elements may provide conflicting cues to ES cells, resulting in inhibition or retardation of their differentiation. Further, non-neural factors such as neo-angiogenesis may be required for the optimal differentiation of these cells.

We have previously shown that CNS-derived NS grafted in the pylorus of mice generate neurons and glia and partially restore gastric function [7]. We here show that ES-derived neurospheres do not compare to CNS-NS after transplantation. Clearly, there is a lot more to be done in order to fully understand the mechanisms for this different behavior. It is possible that the lack of GFAP expression (and glia?) accounts for the poor outcome *in vivo*. Further work will need to be done to address this question.

In this regard, our study had several limitations. First, it should be noted that the time frame of these experiments was relatively short. Long-term survival and differentiation of grafted cells needs to be investigated in further studies to fully assess the potential of ES-NS to form enteric neurons *in vivo*. It is also possible that “priming” of the ES, perhaps by exposure to LM-MP prior to the transplantation may result in a better outcome. Finally, the inability to precisely localize the injection into the muscle layer may be a factor in preventing the appropriate signals from reaching the ES cells (in our study most of them were found in the submucosa). Therefore, this study cannot confidently exclude the therapeutic potential of ES cell for repopulating the ENS. On the other hand, if they truly do not differentiate, they may retain the ability to proliferate (which we did not examine), which is of concern because of the risk of tumorigenesis.

## Conclusions

A variety of cell sources are currently being explored for use in cell transplantation studies including CNS-derived NS, neural crest stem cells (NCCS), enteric neural progenitor cells (ENPC) and embryonic stem cells (ES) [5,42]. Among these sources, ES cells have the advantage of being available in large numbers and of being capable to adopt a full spectrum of cell fates. In this study we have shown that ES-derived neurospheres (ES-NS) are able to generate specific phenotypes under the influence of diffusible factors *in vitro*, while they fail to differentiate after transplantation in the gut. The inability of ES-NS to adopt a neuronal phenotype *in vivo* might be due to local inhibitory influences that prevents their differentiation, and suggests that ES-NS may not be suitable candidates for ENS regeneration.

## Competing interests

The author(s) declare that they have no competing interests.

## Authors’ contributions

VS carried out the experiments, performed the statistical analysis and drafted the manuscript. MAM participated in the design of the study, in the statistical analysis and in the drafting of the manuscript. KMK performed cell counting and participated in the cell transplantation experiments. PJP conceived the study, and participated in its design and coordination and helped to draft the manuscript. All authors read and approved the final manuscript.

## Authors’ information

The current affiliation of VS is Division of Molecular Neurobiology, MRC National Institute for Medical Research, The Ridgeway, Mill Hill London NW71AA, UK.

The current affiliation of MAM is Department of Anesthesiology, University of Texas Medical Branch, Galveston, TX, USA.

The current affiliation of PJP is Johns Hopkins Center for Neurogastroenterology, Johns Hopkins University School of Medicine, Baltimore, MD, USA.

## Pre-publication history

The pre-publication history for this paper can be accessed here:

http://www.biomedcentral.com/1471-230X/12/81/prepub
